# Pharmacovigilance analysis of infliximab in inflammatory bowel disease: novel safety signals and sex-specific adverse event profiles from the FAERS database (2004–2024)

**DOI:** 10.3389/fimmu.2026.1662405

**Published:** 2026-03-24

**Authors:** Wei Liu, Shaochen Wang, Gang Wang, Zhenguo Qiao, Li Rao, Long Cheng, Genhai Shen

**Affiliations:** 1 Department of Minimally Invasive Common Surgery, Suzhou Ninth People’s Hospital, Xuzhou Medical University Suzhou Bay Clinical College, Suzhou, Jiangsu, China; 2 Department of Interventional Radiology, Dushu Lake Hospital Affiliated to Soochow University, Medical Center of Soochow University, Suzhou Dushu Lake Hospital, Suzhou, Jiangsu, China; 3 Department of Gastroenterology, Suzhou Ninth People’s Hospital, Xuzhou Medical University Suzhou Bay Clinical College, Suzhou, Jiangsu, China; 4 Department of Geriatrics, Renmin Hospital of Wuhan University, Wuhan, Hubei, China

**Keywords:** Crohn’s disease, disproportionality analysis, FAERS, inflammatory bowel disease, infliximab, pharmacovigilance, ulcerative colitis

## Abstract

**Introduction:**

Inflammatory bowel disease (IBD) is a chronic immune-mediated gastrointestinal disease, and its global incidence is on the rise, which seriously affects the quality of life of patients. Infliximab is the key therapeutic drug for IBD, and a comprehensive safety assessment is needed. In this pharmacovigilance study, we investigated the adverse events (AE) of infliximab in IBD patients by analyzing the reports submitted to the FDA’s Adverse Event Reporting System (FAERS) database.

**Methods:**

We analyzed the reports of AEs related to infliximab in FAERS database (2004-2024). Disproportionality analysis (ROR, PRR, BCPNN) was used to identify the safety signals in the general population and gender subgroups. Based on the model of ROR, the influence of gender difference and combined medication was evaluated. The onset time (TTO) and Weibull shape parameter (WSP) were used to analyze and evaluate the occurrence time and risk trend of AEs.

**Results:**

Among 80,138 AE reports, 57 Preferred Term (PT) and 14 System Organ Classification (SOC) signals were detected (ROR025 > 1, PRR > 2, χ² > 4, N ≥ 3, IC025 > 0). Some emerging AE signals, such as Horner’s syndrome and Henoch-Schoenlein Henoch-Schonlein purpura nephritis (not mentioned in the drug label), suggest that there are new safety hazards. Females (50.7%) exhibited 67 signals, predominantly immune-related (e.g., lupus-like syndrome, N = 1028, ROR = 7.09), while males (41.8%) showed 42 signals, mainly cardiovascular (e.g., blood pressure fluctuation, ROR = 29.33) and infectious. Combined medication (67.3%) will increase cardiovascular risk, while monotherapy is associated with immune/tumor-related AEs (such as breast cancer, ROR = 1.2). Kidney and urinary system diseases (ROR = 21.84) are an under-reported problem. Time trade-off analysis (TTO, N = 15,682) showed that the median treatment duration was 620 days, and the early treatment failed (Weibull β=0.73). In addition, there was a significant gender difference in the incidence of AEs related to infliximab in IBD patients.

**Conclusion:**

This study emphasizes that there is a significant burden of AEs in IBD patients treated with infliximab, and finds new safety signals that need further verification. The characteristics of gender-specific AEs suggest that gender-specific monitoring is needed. Women have a higher risk of immune/tumor events, while men have a higher risk of cardiovascular/infectious AEs. Combined medication will aggravate cardiovascular risk, while monotherapy will increase immune/carcinogenic risk. The occurrence of early AEs highlights the necessity of early close monitoring. These findings suggest that pharmacovigilance needs to be improved to optimize the safety of infliximab.

## 1 Introduction

Inflammatory bowel disease (IBD) is a chronic recurrent gastrointestinal disease caused by immune dysregulation, including Crohn’s disease (CD) and ulcerative colitis (UC). Typical clinical manifestations include abdominal pain, diarrhea, mucus and blood in the stool and weight loss, usually accompanied by various parenteral symptoms. These symptoms seriously affect the quality of life of patients and usually require lifelong treatment and management. IBD affects about 4.9 million people worldwide, with the highest number of cases in China and the United States in 2019. The global age-standardized incidence increased slightly from 4.22 cases per 100,000 people in 1990 to 4.45 cases per 100,000 people in 2021, with a prevalence rate of 59.3 cases per 100,000 people, and the crude prevalence rate increased by 47%. In newly industrialized countries, such as Asian and Latin American countries, especially China and India, the incidence rate has increased significantly (1). Traditional therapies mainly include aminosalicylate, corticosteroids and immunomodulators; Biotherapy, especially anti-tumor necrosis factor-α (TNF-α) preparation, has become the cornerstone of treatment for moderate and severe cases or patients who do not respond to standard treatment (2).

Infliximab is a chimeric monoclonal antibody targeting TNF-α, which was approved by FDA in 1998 for the treatment of moderate and severe active Crohn’s disease, and in 2005 for the treatment of moderate and severe ulcerative colitis, becoming the first biological agent widely used in the treatment of IBD (3). Infliximab inhibits the interaction between TNF-α and its receptor by specifically binding to it, thus inhibiting the inflammatory cascade, alleviating intestinal inflammation and promoting mucosal repair (4). A large number of randomized controlled trials (RCT) confirmed its efficacy in inducing and maintaining remission of IBD (3–8). However, infliximab can also cause some adverse reactions. Common adverse reactions include infusion-related reactions (such as fever, chills and rash), upper respiratory tract infection, headache, joint pain and gastrointestinal discomfort, and the incidence rate is about 10-20% (3, 4, 6, 9). In addition, due to its immunosuppression, patients may face an increased risk of serious infections (such as tuberculosis, invasive fungal infections and viral infections) and malignant tumors (such as non-Hodgkin’s lymphoma). Therefore, comprehensive screening is needed before treatment and regular monitoring is needed during treatment (10). With the expansion of clinical application, real-world studies have reported new or rare adverse reactions related to infliximab, including demyelination of the central nervous system, lupus-like syndrome and heart failure (11). In addition, the incidence of delayed hypersensitivity and immune complex-mediated adverse events (AE) (such as serum sickness-like reaction and rheumatoid arthritis) has also increased, so clinicians and patients need to be vigilant (12). Despite these safety problems, infliximab is still the key drug to treat IBD, especially for patients with hormone dependence or refractory hormone. Compared with other anti-TNF-α drugs (such as adalimumab and golimumab), its efficacy is relatively stable (13).

In clinical trials, rare or emerging adverse reactions are often difficult to identify comprehensively, which highlights the urgent need for large-scale and systematic pharmacovigilance data for in-depth investigation. This study leverages the U.S. FDA Adverse Event Reporting System (FAERS), a well-established spontaneous reporting database widely utilized for post-marketing drug safety assessments (14). We aim to employ non-proportional analysis techniques for detecting infliximab-related adverse event signals, utilizing established frequency-based metrics, including the Reporting Odds Ratio (ROR), Proportional Reporting Ratio (PRR), and Bayesian Confidence Propagation Neural Network (BCPNN). The aim is to systematically identify common and rare adverse reaction signals potentially associated with IBD patients, in order to provide evidence-based guidance for clinical drug use and enhance the early warning capability for drug safety risks (15).

## 2 Materials and methods

### 2.1 Data source and study design

To obtain pharmacovigilance data on infliximab in IBD patients, we conducted a retrospective analysis of the FAERS database (https://fis.fda.gov//extensions/FPD-QDE-FAERS/FPD-QDE-FAERS.html). The FFAERS database is updated quarterly and includes seven primary datasets: demographic and administrative information (DEMO), drug information (DRUG), adverse event information (REAC), patient outcome data (OUTC), report source (RPSR), therapy start and end dates (THER), and drug indication data (INDI). In the DRUG dataset, each drug’s role in a given report is indicated by specific codes: PS (primary suspect drug), SS (secondary suspect drug), C (concomitant drug), and I (interacting drug) (16).

Given the presence of duplicate reports in the FAERS database, data cleaning was performed in accordance with the FDA’s recommended deduplication criteria to improve data quality and ensure the reliability of study results: when the CASEID was identical, the report with the most recent FDA_DT (date the report was received) was retained; if both CASEID and FDA_DT were the same, the record with the larger PRIMARYID was kept (17).

We retrieved AE reports involving the use of infliximab in the treatment of IBD from the first quarter of 2004 to the fourth quarter of 2024. The identification of cases of IBD is based on the INDI field in FAERS database, and only reports that explicitly list Crohn’s disease, ulcerative colitis or inflammatory bowel disease as the main indications are included. In order to minimize the potential classification deviation, the reports that the indications were unclear or that IBD was not clearly pointed out as the main therapeutic indication were excluded. The generic name and trade name of infliximab (for example, Remicade) were used in the retrieval process, and only the report that infliximab was designated as the main drug was retained to enhance the causal relationship between drug exposure and AEs.

MedDRA is a global standardized medical terminology system, which aims to ensure the consistency of recording and reporting adverse event data in international pharmacovigilance. It adopts a five-level hierarchical structure, including the lowest level term (LLT), preferred term (PT), advanced term (HLT), advanced group term (HLGT) and system organ classification (SOC) (18). In FAERS database, all adverse event (AE) data are coded by MedDRA, which is a key process to improve the uniformity and accuracy of pharmacovigilance analysis. In this study, we used the PT grade of MedDRA 27.1 to classify and evaluate AE, focusing on accurately identifying the AE reports related to infliximab on SOC and PT grade.

### 2.2 Statistical analysis

Disproportionality analysis is a cornerstone statistical method in pharmacovigilance, which aims to evaluate the intensity of association between specific drugs and AE. We used three mature signal detection methods-ROR, PRR and BCPNN-to comprehensively evaluate the potential association between infliximab use and AEs in IBD patients (19).

ROR and PRR are frequency-based proportional imbalance indicators. When the lower limit of 95% confidence interval (CI) of ROR (ROR025) is greater than 1 and the number of related reports is ≥ 3, it is considered that there is a positive signal. Similarly, PRR > 2, χ² > 4 and at least 3 related reports also indicate the existence of potential signals. The higher the ROR or PRR value, the stronger the correlation (20).

BCPNN is a Bayesian-based method, which can effectively correct the report deviation and sample variation, thus improving the reliability of signal detection. This method uses information component (IC) as an index; When the lower limit of 95% confidence interval (IC 025) is greater than 0, it is defined as a positive signal. The higher the IC value, the stronger the correlation. This method is more robust in dealing with sparse data and heterogeneous reports (21). **Tables 1** and **2** list the 2×2 contingency table, calculation formula and signal detection threshold of each method in detail.

**Table 1:** 2×2 Contingency table for disproportionality analysis and signal detection criteria.

Item	Number of target adverse event reports	Number of other adverse event reports	Total
Target Drug	A	B	A + B
Other Drugs	C	D	C + D
Total	A + C	B + D	N=A + B + C + D

A, number of reports containing both the target drug and target adverse reaction reports; B, number of reports containing other adverse reaction reports of the target drug; C, number of reports containing the target adverse reaction reports of other drugs; D, number of reports containing other drugs and other adverse reaction reports; N, the number of reports.

**Table 2:** Summary of three signal detection algorithms and their thresholds.

Method	Formula	Threshold
ROR	ROR = ad/bc	lower limit of 95% CI>1, a≥3
	SE(lnROR) = √(1/a + 1/b + 1/c + 1/d)	
	95%CI = e^(ln(ROR) ± 1.96se)	
PRR	PRR = [a(c + d)]/[c(a + b)]	PRR>2, χ2>4, a≥3
	χ² = (ad - bc)² (a + b + c + d)/[(a + b)(c + d)(a + c)(b + d)]	
BCPNN	IC = log 2a(a + b + c + d)/[(a + c)(a + b)]	IC025>0
	95%CI = E(IC) ± 2[V(IC)]^0.5	

We applied the three methods described above to identify the positive signals of the general population and gender analysis. In order to further explore the distribution characteristics of positive signals in different populations, we constructed an analysis model based on ROR to evaluate gender differences and the impact of combined medication. It is worth noting that the ROR in this study is not a causal indicator in the strict sense, which is different from the formal pharmacoepidemiological study. We mainly use it to identify the frequency trend of specific PT signals in different subgroups (22).

ROR value is calculated based on 2×2 contingency table, and P value is obtained by chi-square (χ²) test. To visually present the analytical results, volcano plots were generated using the “ggplot2” package (version 3.3.6) in R, where the x-axis represents the log-transformed ROR (logROR), and the y-axis denotes the FDR-adjusted -log_10_(P value) (P.adj). When ROR > 1 and P.adj < 0.05, it suggests that the AE is more likely to occur in females or individuals not using concomitant medications. Conversely, when ROR < 1 and P.adj < 0.05, the event is more likely to occur in males or individuals receiving concomitant medications (23).

### 2.3 Time to onset analysis of AEs

We calculated the time to onset (TTO) of AEs, defined as the time interval between the event occurrence date (EVENT_DT) and the start date of infliximab administration (START_DT) (24). To keep the analysis accurate, we removed reports with clear data errors — such as EVENT_DT earlier than START_DT, incomplete or unreliable date information, and those missing essential variables. The statistical analysis of TTO was conducted using the median, interquartile range, and Weibull shape parameter (WSP) test (25, 26).

The WSP test was applied to evaluate the time-dependent risk trend of AE occurrence. The Weibull distribution is characterized by two parameters: the scale parameter (α) and the shape parameter (β) (25, 26). To assess whether the risk of infliximab-related AEs increased or decreased over the course of treatment, we calculated the median TTO and the corresponding WSP values (25, 26). All WSP analyses were performed using R Studio software (version 4.3.3).

## 3 Results

### 3.1 Characteristics of reported cases

**Figure 1:**
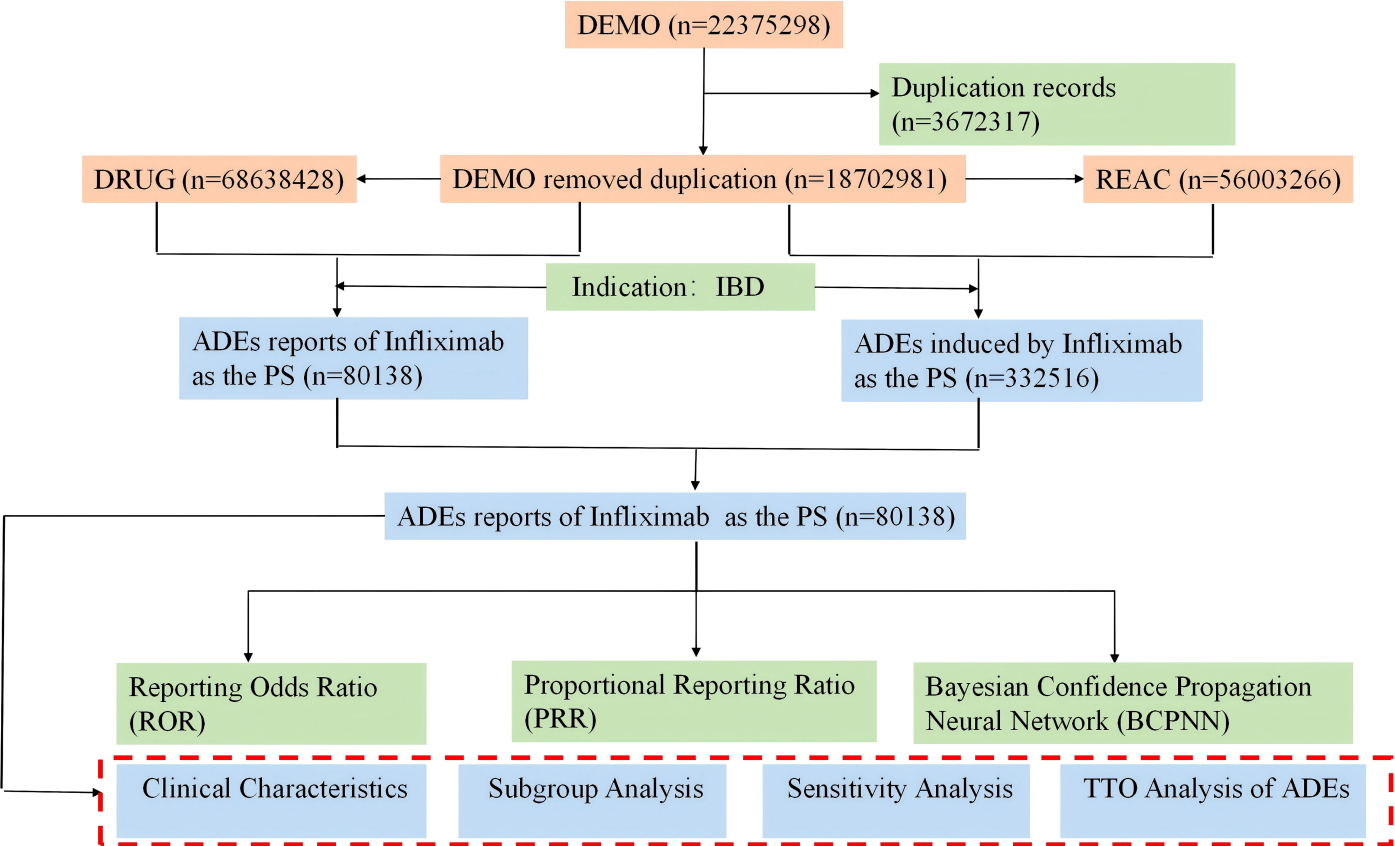
The process of identifying adverse events associated with Infliximab in IBD patients using data from the FAERS database.

From the first quarter of 2004 to the fourth quarter of 2024, a total of 80,138 AE reports related to infliximab use in IBD patients were retrieved from the FAERS database after data processing. The detailed extraction process is illustrated in **Figure 1**. We conducted statistical analysis and visualization of these reports.

Regarding gender distribution, female patients accounted for a higher proportion (50.7%) compared to male patients (41.8%). The majority of reports originated from Canada (48.7%), followed by the United States (45.8%), Brazil (3.4%), France (2.8%), Japan (2.4%), and the United Kingdom (2.2%). Most adverse drug event reports were submitted by healthcare professionals. Detailed demographic and baseline information is summarized in **Table 3**.

**Table 3:** Clinical characteristics of reports involving Infliximab in patients with IBD from the FAERS database.

Characteristics	Infliximab-Induced ADE (N = 80138)
Age	
≤17 years	11644 (14.5%)
18˜64 years	42035 (52.5%)
65˜85 years	6096 (7.6%)
≥86 years	187 (0.2%)
Missing	20176 (25.2%)
Gender	
Female	40658 (50.7%)
Male	33535 (41.8%)
Missing	5945 (7.5%)
Weight	
<50 kg	6988 (8.7%)
50˜100 kg	36271 (45.3%)
>100 kg	4270 (5.3%)
Missing	32609 (40.7%)
Occupation of the reporter	
Pharmacist (PH)	1942 (2.4%)
Consumer (CN)	17780 (22.2%)
Lawyer (LW)	41 (0.1%)
Medical Doctor (MD)	20354 (25.4%)
Other Health-Professional (OT)	19132 (23.9%)
Health Professional (HP)	20588 (25.7%)
Registered Nurse (RN)	5 (0%)
Missing	296 (0.4%)
Country of the reporter	
Canada	39022 (48.7%)
United States	20693 (45.8%)
Brazil	2777 (3.4%)
France	2188 (2.8%)
Japan	1950 (2.4%)
United Kingdom	1770 (2.2%)
Germany	1363 (1.7%)
Australia	1133 (1.4%)
Italy	833 (1.0%)
Spain	760 (0.9%)
Netherlands	505 (0.7%)
Belgium	473 (0.6%)
Other countries	5167 (6.4%)
Missing	1504 (1.8%)

**Figure 2:**
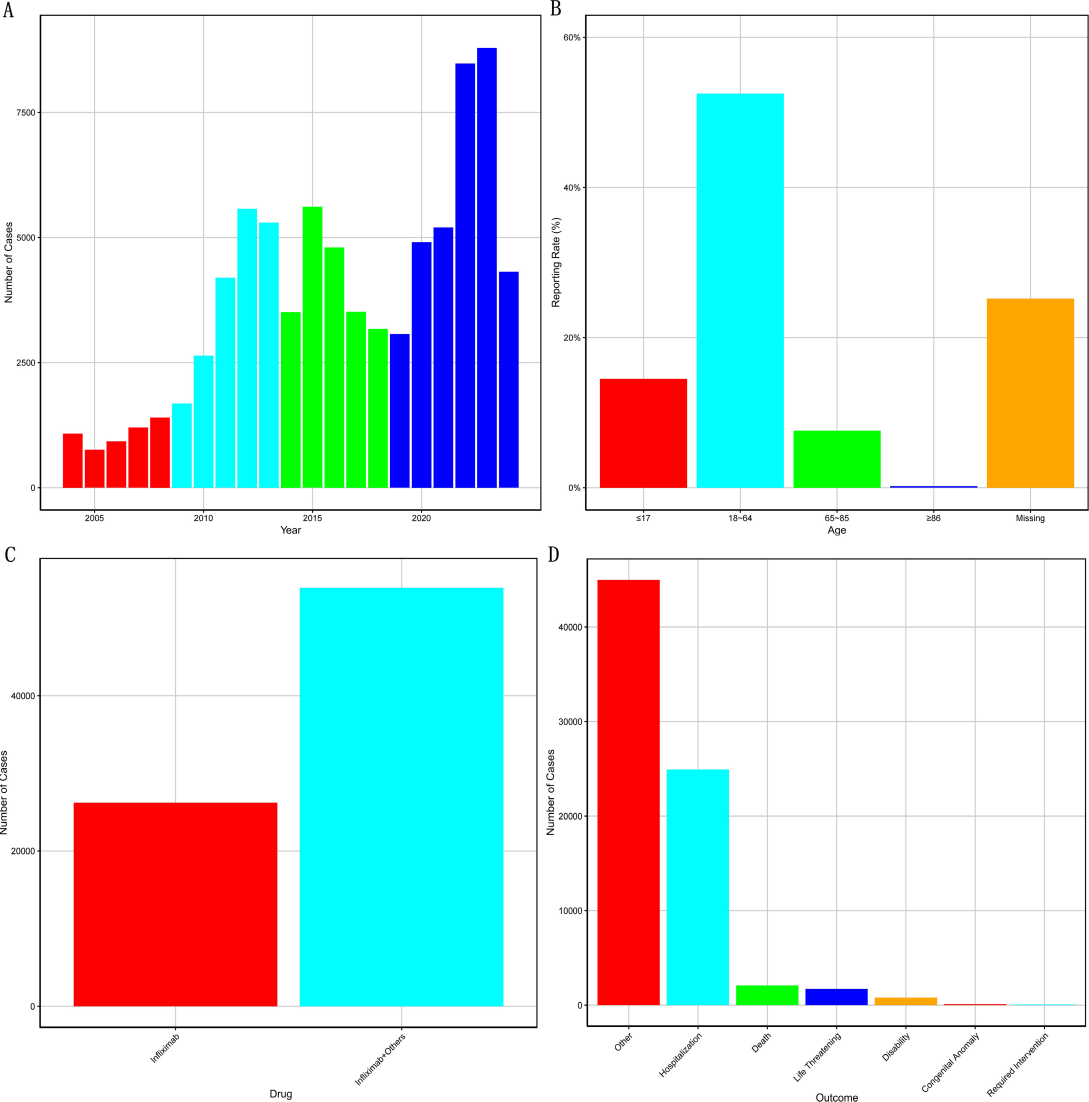
Statistical analysis of infliximab-related adverse events in IBD Patients from the FAERS Database. **(A)** Annual Distribution of Infliximab-Related Adverse Event Reports in IBD patients; **(B)** Reporting Rates of Infliximab-Related Adverse Events in IBD patients Stratified by Age; **(C)** Concomitant Medications Associated with Infliximab-Related Adverse Events in IBD patients; **(D)** Clinical Outcomes of Adverse Reactions Linked to Infliximab-Related Adverse Events in IBD patients.

In IBD cases, the number of infliximab-related AE reports showed a fluctuating upward trend, reaching a peak in 2023 with a total of 8,788 reports (**Figure 2A**). In terms of age at onset (**Figure 2B**), the 18–64 age group accounted for the highest proportion (52.5%), followed by patients under 18 years (14.5%), those aged 65–85 years (7.6%), and those older than 85 years (0.2%).

Regarding concomitant medications (**Figure 2C**), 26,228 (32.7%) IBD patients were treated with infliximab alone, while 53,910 (67.3%) received infliximab in combination with other drugs. The most commonly co-administered medications included prednisone, azathioprine, methotrexate, mesalamine, and humira.

As for clinical outcomes (**Figures 2D**, with the most severe outcome selected per individual), the most frequently reported outcome was “Other Serious – Medically Important Condition” (44,968 cases), followed by hospitalization (24,908), death (2,080), life-threatening events (1,714), disability (796), intervention required to prevent permanent damage (114), and congenital anomaly (61).

### 3.2 Signal detection of SOC levels for infliximab in IBD patients

**Figure 3:**
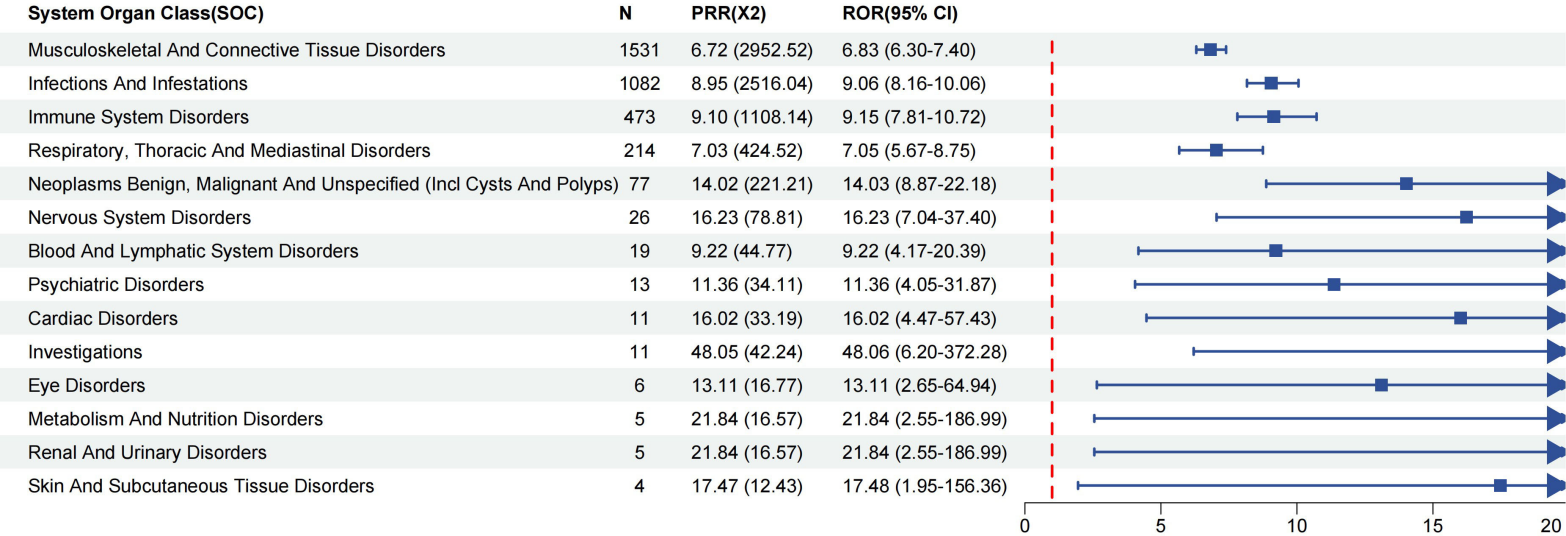
Signal strength of infliximab-associated adverse event reports in IBD Patients at the SOC Level. ROR, Reporting Odds Ratio; PRR, Proportional Reporting Ratio.

A statistical analysis of dose-related SOCs associated with infliximab in IBD patients revealed 14 affected SOCs (**Figure 3**). Based on the ROR025 > 1, PRR > 2, χ² > 4, N ≥ 3, the most strongly associated SOC was investigations (ROR = 48.06, PRR = 48.05, χ² = 42.24), followed by metabolism and nutrition disorders (ROR = 21.84, PRR = 21.84, χ² = 16.57) and renal and urinary disorders (ROR = 21.84, PRR = 21.84, χ² = 16.57). Renal and urinary disorders has not been adequately addressed in the drug label, suggesting a potential safety concern.

In terms of case numbers, the most common SOCs were musculoskeletal and connective tissue disorders (N = 1531), followed by infections and infestations (N = 1082) and immune system disorders (N = 473). These findings highlight the key areas of AEs related to infliximab and provide important evidence for clinical risk management of IBD patients. It is worth noting that although some SOCs, such as kidney and urinary system diseases, show strong signal values, the stability or reliability of the signal may be reduced due to the small number of cases (N = 5). On the other hand, some high-frequency SOCs, such as infections and parasitic diseases (N = 1082), only have moderate signal intensity (ROR = 9.06, PRR = 8.95, χ² = 2516.04), which does not reach the higher threshold we usually seek. This indicates that the clinical relevance of these signals may need more data for further verification. Future research is recommended to expand sample size and incorporate clinical context, patient characteristics, and mechanistic studies to clarify the clinical significance of these signals and enhance safety management of infliximab in IBD patients.

### 3.3 Signal detection at the PT level for infliximab in IBD patients

**Figure 4:**
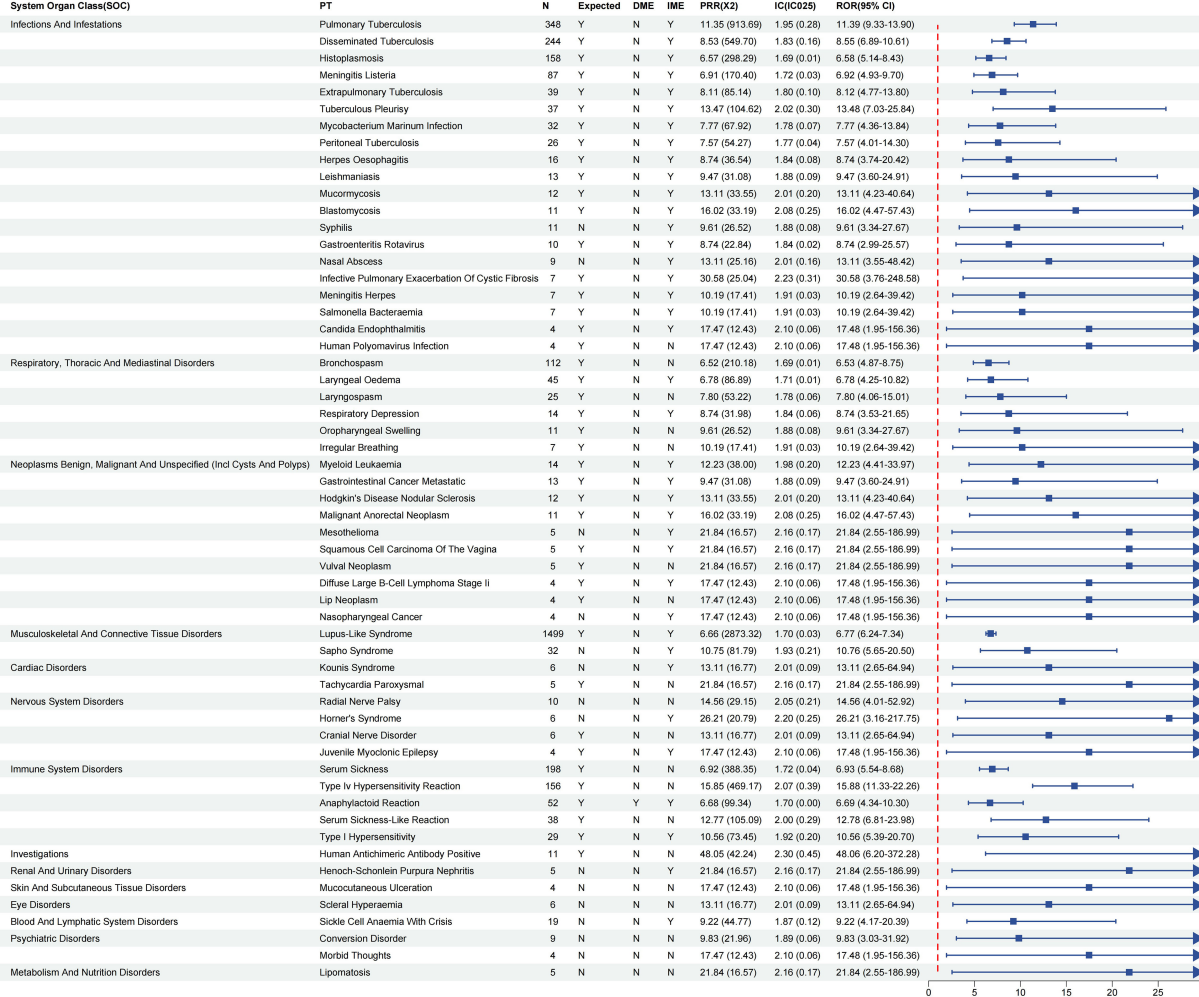
Signal strength of infliximab-associated adverse event reports in IBD Patients at the PT Level. ROR, Reporting Odds Ratio; PRR, Proportional Reporting Ratio; IC, Information Component; IC025, Lower Limit of 95% CI of IC.

Using the FAERS database, a signal detection analysis of AEs related to infliximab in IBD patients identified 57 signals at the PT level (**Figure 4**). The inclusion criteria for signal detection were: ROR025 > 1, PRR > 2, χ² > 4, N ≥ 3, and IC025 > 0. Signals meeting these criteria were subjected to further analysis.

Among PTs with more than 100 reports, the most frequently reported AEs related to infections and infestations were pulmonary tuberculosis (n = 348), disseminated tuberculosis (n = 244), and histoplasmosis (n = 158); In the category of musculoskeletal and connective tissue disorders, lupus-like syndrome was frequently reported (n = 1499); bronchospasm (n = 112) was the predominant event under respiratory, thoracic and mediastinal disorders; Within immune system disorders, serum sickness (n = 198) and type IV hypersensitivity reaction (n = 156) were particularly notable. Most of these AEs have already been documented in the product label for infliximab.

Further analysis of PTs with high ROR values revealed several AEs with low report counts but strong signal strength, warranting attention. These included henoch-schonlein purpura nephritis (n = 5, ROR = 21.84), mucocutaneous ulceration (n = 4, ROR = 21.84), lip neoplasm (n = 4, ROR = 17.48), nasopharyngeal cancer (n = 4, ROR = 17.48), and morbid thoughts (n = 4, ROR = 17.48). These rare AEs may indicate potential safety concerns that require further investigation and validation.

Ranking all PTs by descending ROR values, the top 10 AEs with the strongest signals were: human antichimeric antibody positive (ROR = 48.06), infective pulmonary exacerbation of cystic fibrosis (ROR = 30.58), horner’s syndrome (ROR = 26.21), henoch-schonlein purpura nephritis (ROR = 21.84), lipomatosis (ROR = 21.84), mesothelioma (ROR = 21.84), squamous cell carcinoma of the vagina (ROR = 21.84), tachycardia paroxysmal (ROR = 21.84), vulval neoplasm (ROR = 21.84), and candida endophthalmitis (ROR = 17.48). Notably, horner’s syndrome, henoch-schonlein purpura nephritis, lipomatosis, and mesothelioma are not currently listed in the infliximab label, suggesting the presence of potential new safety signals.

Overall, the AE signals associated with infliximab were predominantly concentrated in known domains such as infections, immune disorders, and respiratory complications, including pulmonary tuberculosis, lupus-like syndrome, and bronchospasm. However, several high-frequency but undocumented PTs (e.g., henoch-schonlein purpura nephritis, mucocutaneous ulceration), along with low-frequency yet high-signal-strength PTs (e.g., lip neoplasm, nasopharyngeal cancer, morbid thoughts), suggest the existence of unrecognized safety risks that warrant further clinical investigation and validation.

### 3.4 Sex-stratified subgroup analysis

In the pharmacovigilance study of infliximab in IBD patients, AEs were identified separately in male and female subgroups at the PT level. The screening criteria were: ROR025 > 1, PRR > 2, χ² > 4, N ≥ 3, and IC025 > 0.

**Figure 5:**
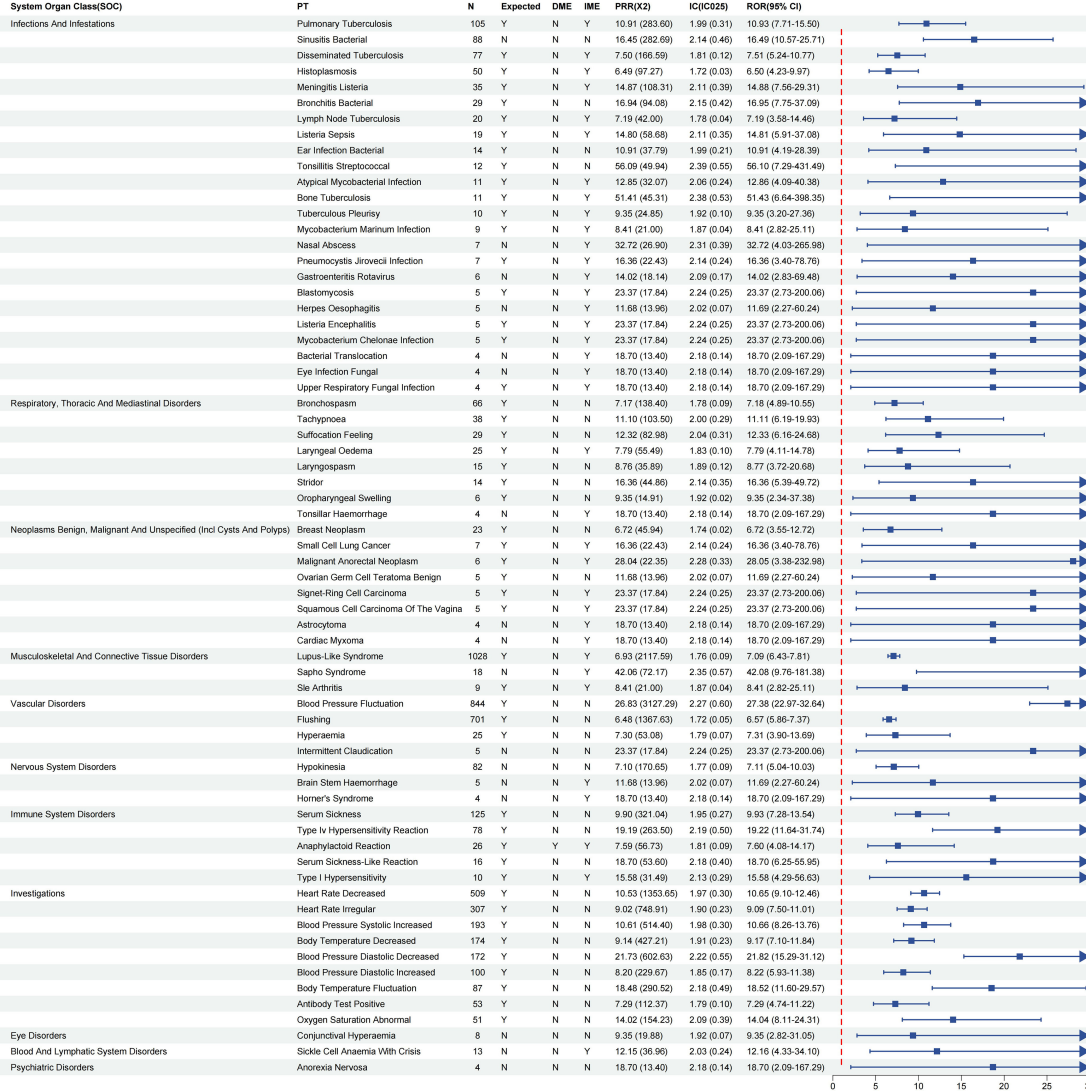
Signal strength of infliximab-associated adverse event reports in Female IBD patients at the PT Level. ROR, Reporting Odds Ratio; PRR, Proportional Reporting Ratio; IC, Information Component; IC025, Lower Limit of 95% CI of IC.

In female IBD patients, a total of 67 potential AEs signals were detected, covering multiple systems including infections, neoplasms, cardiovascular, respiratory, urogenital, immune, and nervous systems (**Figure 5**). Events with high report counts and strong signal intensity included lupus-like syndrome (N = 1028, ROR = 7.09, 95% CI: 6.57–7.65), blood pressure fluctuation (N = 844, ROR = 27.38, 95% CI: 25.73–29.16), and flushing (N = 701, ROR = 6.57, 95% CI: 6.08–7.10), suggesting these events occur more frequently in female patients and are significantly associated with infliximab. Moreover, several events with moderate report counts but very strong signal intensities also warrant attention, such as streptococcal tonsillitis (N = 12, ROR = 56.10, 95% CI: 30.99–101.58), skeletal tuberculosis (N = 11, ROR = 51.43, 95% CI: 28.43–93.06), and SAPHO syndrome (N = 18, ROR = 42.08, 95% CI: 26.79–66.13), indicating that infliximab treatment may trigger serious infections or rare immune-related responses. Notably, although some AEs had relatively low report numbers, the markedly elevated ROR values also suggest potential risks. For example, nasal abscess (N = 7, ROR = 32.72, 95% CI: 14.62–73.27), squamous cell carcinoma of the vagina (N = 5, ROR = 23.37, 95% CI: 7.34–74.28), signet ring cell carcinoma (N = 5, ROR = 23.37, 95% CI: 7.34–74.28), and intermittent claudication (N = 5, ROR = 23.37, 95% CI: 7.34–74.28) suggest that these rare but strongly associated events in the female subgroup merit further monitoring and validation.

**Figure 6:**
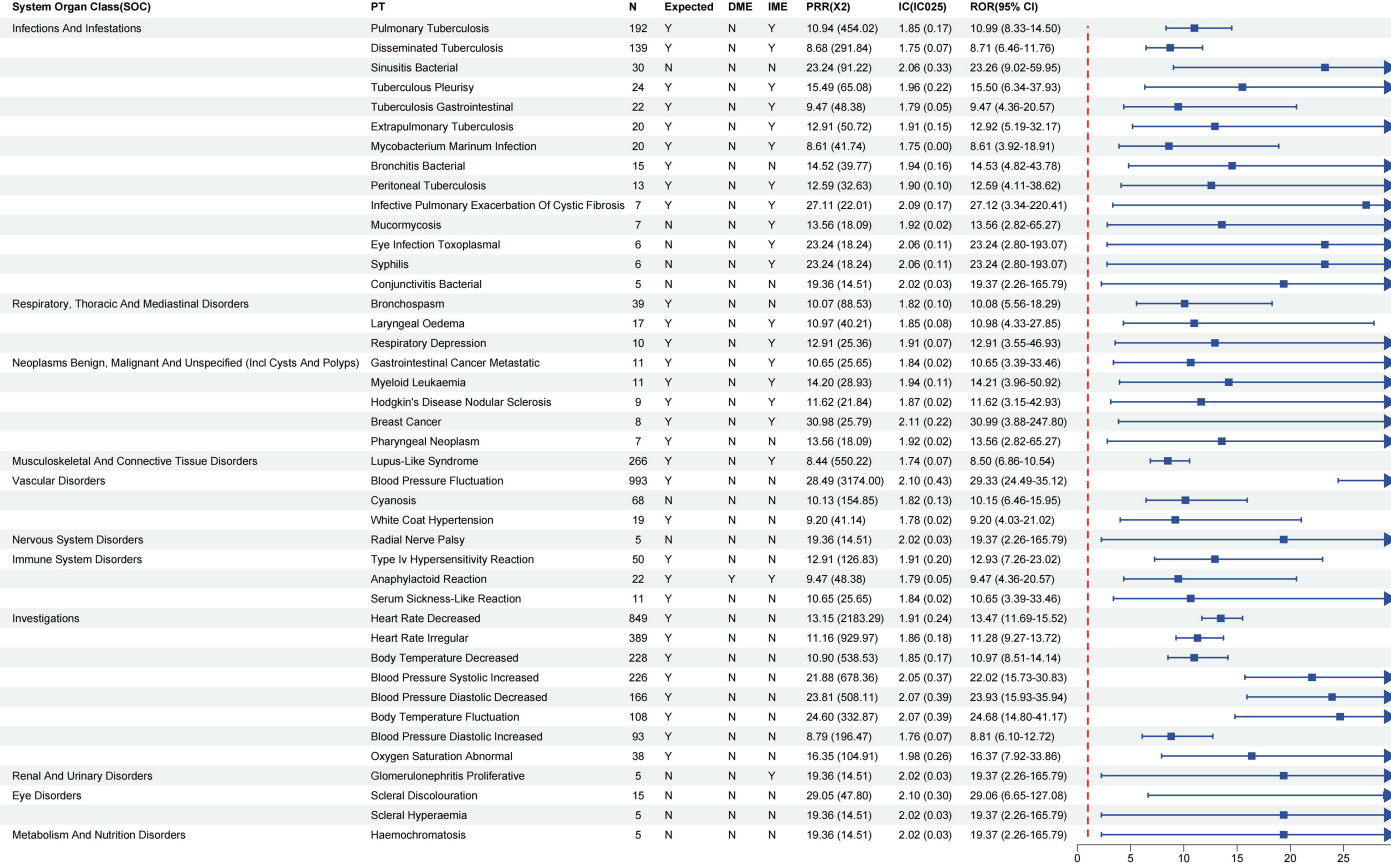
Signal strength of infliximab-associated adverse event reports in Male IBD patients at the PT Level. ROR, Reporting Odds Ratio; PRR, Proportional Reporting Ratio; IC, Information Component; IC025, Lower Limit of 95% CI of IC.

In the male subgroup, we identified 42 potential AE signals affecting various organ systems, such as infections, neoplasms, cardiovascular issues, respiratory problems, urogenital disorders, immune-related events, and nervous system conditions (**Figure 6**). Events such as blood pressure fluctuation (N = 993, ROR = 29.33, 95% CI: 24.49–35.12), bradycardia (N = 849, ROR = 13.47, 95% CI: 11.19–15.32), and arrhythmia (N = 389, ROR = 11.28, 95% CI: 9.13–13.96) stood out with high numbers of reports and strong ROR values. This suggests these AEs may be more commonly reported–and potentially more relevant–among male IBD patients using infliximab. Additionally, AEs with moderate report counts but markedly elevated ROR values also warrant attention. For example, male breast cancer (N = 8, ROR = 30.99, 95% CI: 8.24–92.47) and scleral discoloration (N = 15, ROR = 29.06, 95% CI: 16.85–50.27) are rare but serious reactions, suggesting a possible association between infliximab and sex-specific neoplastic or mucosal system-related events. Notably, some AEs with low report numbers but markedly elevated RORs. These included: radial nerve palsy (N = 5, ROR = 19.37, 95% CI: 2.26–165.79), proliferative glomerulonephritis (N = 5, ROR = 19.37, 95% CI: 2.26–165.79), scleral injection (N = 5, ROR = 19.37, 95% CI: 2.26–165.79), and hemosiderosis (N = 5, ROR = 19.37, 95% CI: 2.26–165.79). Although these events only had 5 reports each, the signals were remarkably strong, indicating the need for enhanced pharmacovigilance and clinical monitoring of such rare events in male patients.

### 3.5 Gender sensitivity analysis

**Figure 7:**
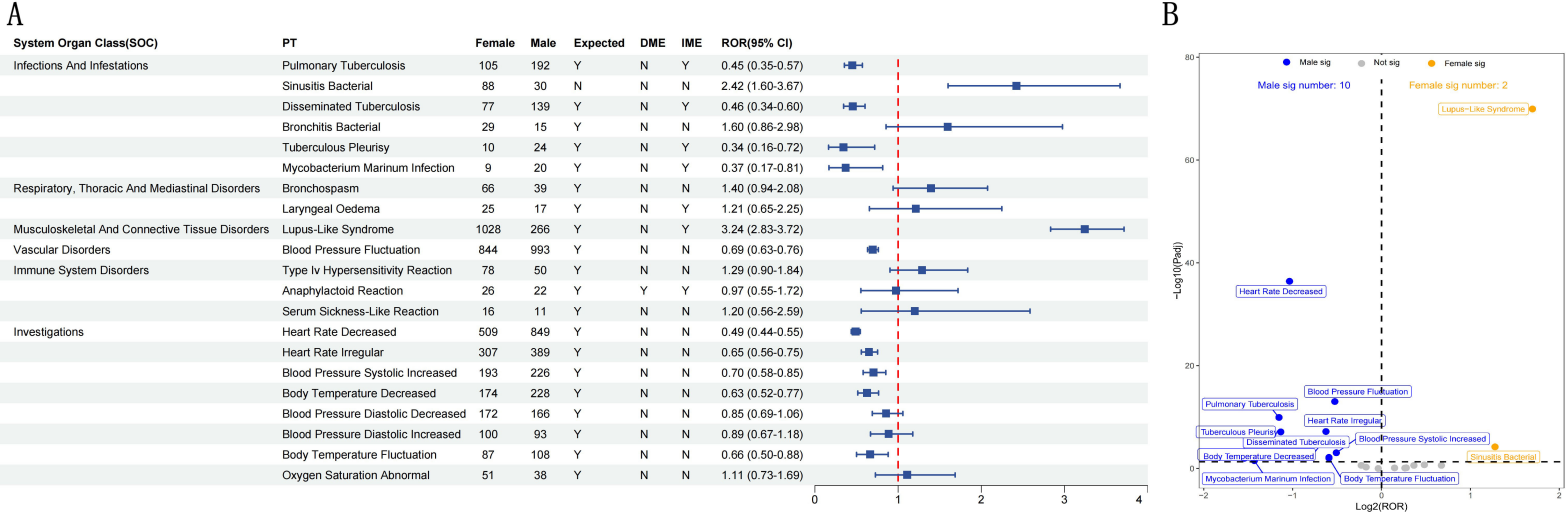
Sex-based sensitivity analysis of 21 positive PT signals in male and female IBD patients. **(A)** Forest plot presenting the Reporting Odds Ratios (RORs) and their corresponding 95% Confidence Intervals (CIs) for all positive PT signals. **(B)** Volcano plot depicting gender-specific differential signals. The x-axis represents the log_2_(ROR) values, and the y-axis shows the –log_10_-transformed adjusted p-values. Statistically significant signals are highlighted in distinct colors and annotated. P-values were adjusted using the False Discovery Rate (FDR) method.

We further analyzed 21 shared positive PT signals between male and female subgroups by using the disproportionality analysis based on ROR method (**Figure 7A**), and visualized the results with volcano diagram (**Figure 7B**). In the volcano map, each point represents a PT. We use blue dots (10 signals) to mark the significant signals of male patients and orange dots (2 signals) to mark the significant signals of female patients. All insignificant signals are displayed in gray.

The significant signals in the male subgroup mainly involve infection and fluctuation of vital signs. For example, tuberculosis, tuberculous pleurisy, Mycobacterium marinum infection and blood pressure fluctuation are statistically significant. This suggests that infliximab may be more likely to induce infection or hemodynamic adverse reactions in male IBD patients. Notably, blood pressure fluctuation ranked among the most frequent shared PT signals in both male and female subgroups. Nevertheless, disproportionality analysis indicated a modestly stronger signal in males (Log_2_[ROR] ≈ -0.53, -Log_10_[Padj] ≈ 13), implying potentially heightened sensitivity or reporting of this adverse event in male patients receiving infliximab. Additionally, signals such as heart rate decreased and body temperature decreased were also observed in the male subgroup, indicating a potential impact on autonomic nervous function following drug administration.

In contrast, the number of significant signals in the female subgroup was lower, with only two signals identified: lupus-like syndrome (Log_2_[ROR] ≈ 1.7, -Log_10_[Padj] ≈ 70) and sinusitis bacterial (Log_2_[ROR] ≈ 1.3, -Log_10_[Padj] ≈ 4.2). Among them, the former showed the strongest significance, suggesting that female IBD patients may be more susceptible to immune-mediated adverse reactions during infliximab treatment.

### 3.6 Concomitant medication sensitivity analysis

**Figure 8:**
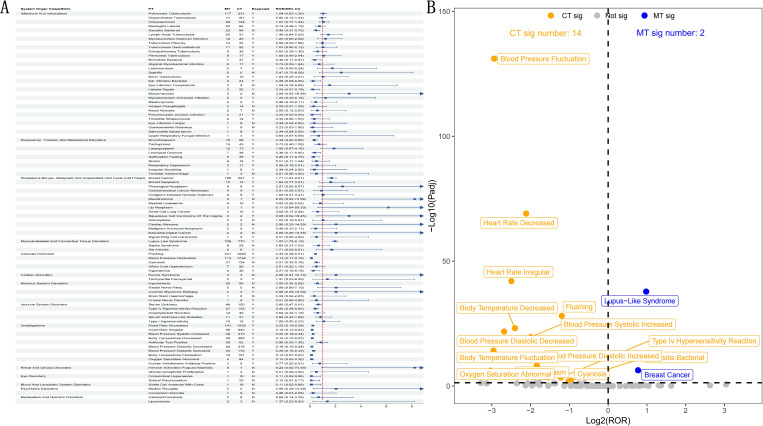
Concomitant medication sensitivity analysis - CT group (concomitant therapy) *vs*. MT group (monotherapy). **(A)** Forest plot presenting the Reporting Odds Ratios (RORs) and their corresponding 95% Confidence Intervals (CIs) for all positive PT signals. **(B)** Volcano plot illustrating signals related to the presence or absence of concomitant medications. The x-axis represents the log_2_(ROR) values, and the y-axis shows the –log_10_-transformed adjusted p-values. Statistically significant signals are highlighted in distinct colors and annotated. P-values were adjusted using the False Discovery Rate (FDR) method.

To further investigated the differences in AEs associated with infliximab treatment in IBD patients with or without concomitant medications, we used the ROR method for disproportionality analysis (see **Figure 8A**) and showed the results in a volcano plot (**Figure 8B**). In the volcano plot, each dot stands for one PT. We highlighted the significant signals in the CT group with orange dots (14 signals) and those in the MT group with blue dots (2 signals). All the non-significant signals appear in gray.

In the CT group, most of the significant signals pointed to cardiovascular and autonomic dysfunction. These included: blood pressure fluctuation (Log_2_(ROR) ≈ -2.9, -Log_10_(Padj) ≈ 131), heart rate decreased, heart rate irregular, body temperature decreased, cyanosis, and flushing. Abnormalities in systolic and diastolic blood pressure as well as oxygen saturation were observed, suggesting an increased risk of circulatory and respiratory AEs with concomitant medication. In addition, immune-related events, such as type IV hypersensitivity reaction, also showed significant signals in this group.

In contrast, the MT group showed only two significant PT signals: lupus-like syndrome (Log_2_(ROR) ≈ 0.98, -Log_10_(Padj) ≈ 38) and breast cancer (Log_2_(ROR) ≈ 0.77, -Log_10_(Padj) ≈ 6), both linked to immune dysregulation or oncogenic potential. This suggests that, without concomitant medications, infliximab may predominantly contribute to immune-mediated disorders or latent malignancies.

### 3.7 Time-to-onset analysis of AEs

In this study, 15,682 valid reports were included for analysis after excluding records with obvious data entry errors (e.g., EVENT_DT earlier than START_DT), inaccurate date information or missing key variables.

**Figure 9:**
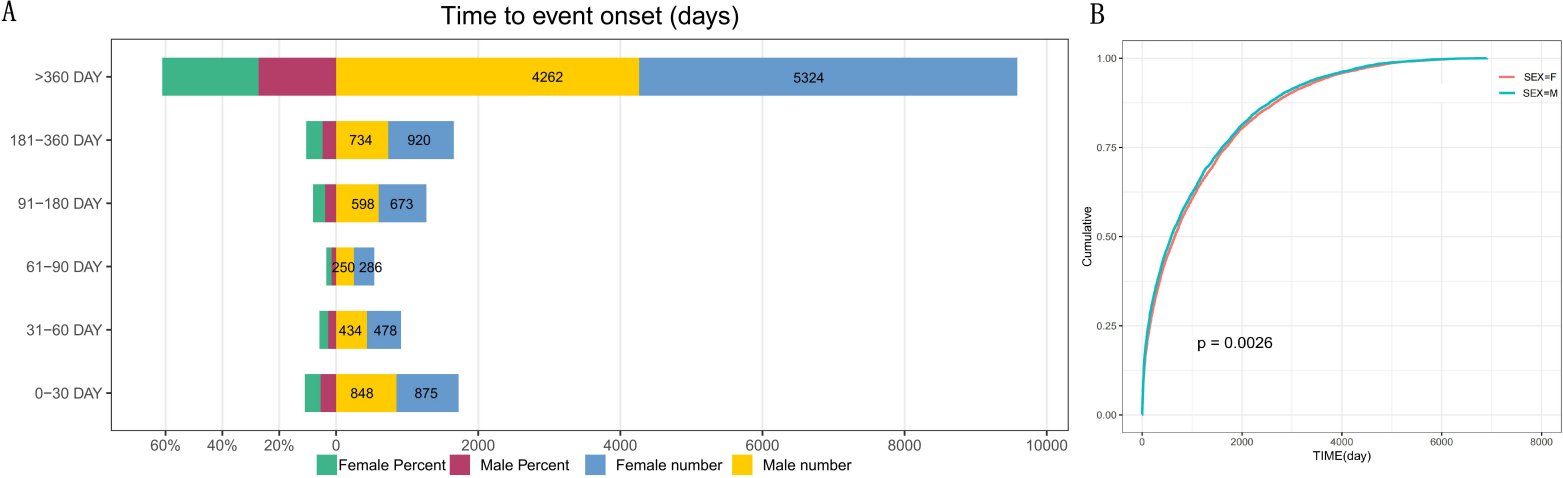
Time to event onset and cumulative incidence analysis by sex in IBD patients. **(A)** Distribution of time to event onset (days) with female and male percentages and numbers across different time intervals. **(B)** Cumulative incidence curves comparing female and male patients, with a log-rank test p-value of 0.0026.

The median treatment interval (TTO) was 620 days and the interquartile interval (IQR) was 1,506 days. The shortest and longest TTOs were 1 day and 6,909 days, respectively. To assess the temporal pattern of AE risk, a WSP test was conducted. The estimated scale parameter (α) was 912.75 (95% CI: 892.19–933.31), and the shape parameter (β) was 0.73 (95% CI: 0.72–0.74) (**Table 4**, **Figure 9A**). As the β value was less than 1, this indicates an early failure distribution pattern, suggesting that the risk of infliximab-related AEs is higher during the early period following drug initiation. Moreover, a significant sex-based difference in infliximab-related event onset was observed among IBD patients (**Figure 9B**).

**Table 4:** Time to onset of infliximab-related adverse events in patients with IBD.

Cases	TTO (days)		Weibull distribution				Failure type
			Scale parameter		Shape parameter		
*n*	Median (IQR)	Min–max	α	95% CI	β	95% CI	
15682	620 (1506)	1-6909	912.75	892.19-933.31	0.73	0.72-0.74	Early failure

n, number of cases with available time-to-onset; IQR, interquartile range; TTO, Time-to-onset.

## 4 Discussion

We analyzed 80,138 reports of infliximab-related AEs in IBD patients from the FAERS database. The results showed a heavy burden of AEs. Women reported a higher proportion (50.7%), and people aged 18–64 accounted for 52.5%. The higher incidence of AEs in women is consistent with the known pattern of IBD-women are usually more susceptible (27). Most reports came from Canada (48.7%) and the United States (45.8%). This may reflect the higher use rate of infliximab or the better reporting system in these countries. The number of AEs showed an overall upward trend. Although it fluctuated, it reached a peak of 8788 cases in 2023. This indicates that infliximab has been widely used in clinic, and it also suggests that its safety needs to be continuously monitored. Clinicians should be aware of this trend and consider close monitoring for AEs, particularly among high-risk groups such as females and middle-aged patients. However, the signals we identified do not prove that infliximab causes these events.

At the SOC level, we found the strongest signals in investigations (mainly positive human antichimeric antibody tests), metabolic and nutritional disorders, and renal and urinary disorders. The prominent signal for renal and urinary disorders does not appear well covered in the current infliximab drug label. This points to a possible new safety concern that needs more study. These associations are hypothesis-generating and cannot be interpreted as confirmed drug-related effects (28, 29). High-frequency SOCs such as musculoskeletal and connective tissue disorders (N = 1,531) and infections and infestations (N = 1,082) are consistent with known risks of infliximab, including lupus-like syndrome and opportunistic infections (30). However, despite their high frequency, the signal strength for infections and infestations was relatively low (ROR = 9.06), which may be confounded by the progression of IBD or concomitant immunosuppressive therapy. Clinicians should regularly monitor for signs of infection and musculoskeletal symptoms, particularly in patients receiving long-term infliximab therapy.

At the PT level, a total of 57 infliximab-related AE signals were identified from the FAERS database. Common signals were mainly related to infections and immune-mediated events, such as pulmonary tuberculosis, disseminated tuberculosis, lupus-like syndrome, and serum sickness, which align with the mechanism whereby infliximab suppresses TNF-α, thereby increasing susceptibility to infections and immune-mediated responses (31). Most of these events have been documented in the drug label and are consistent with findings from previous literature (30).

However, several rare but strong-signal PTs—such as horner’s syndrome, henoch–schonlein purpura nephritis, mesothelioma, lip Tumor, morbid thoughts, and SAPHO syndrome—are not mentioned in the current drug label, indicating potential emerging safety concerns. Unlike the commonly reported infectious and immune events, these signals involve the nervous, renal, neoplastic, psychiatric, and skeletal systems, potentially linked to off-target effects of TNF-α inhibition (32). Among these, henoch–schonlein purpura nephritis represents a key PT with a relatively strong signal under the renal and urinary disorders SOC and is not included in the current prescribing information for infliximab, warranting particular attention. However, it should be emphasized that IBD itself is associated with a spectrum of renal manifestations, with an estimated incidence of 4%-23%. Common presentations include nephrolithiasis (the most frequent), IgA nephropathy/mesangial proliferative glomerulonephritis, tubulointerstitial nephritis, and secondary amyloidosis, with nephrolithiasis and IgA-related nephropathy being especially prominent (33). Therefore, the renal signal found in this analysis may partly reflect the inherent extraintestinal renal involvement of IBD. However, the relatively strong correlation observed in the case of infliximab exposure indicates that inhibition may further undermine immune homeostasis on the basis of disease-related baseline susceptibility. From the mechanism point of view, TNF-α blocking may interfere with mucosal immune regulation and immune complex clearance, which may promote IgA1 glycosylation abnormality or increase glomerular immune complex deposition, and further aggravate renal injury (28, 29). This finding is not an isolated phenomenon, but may reflect a broader immune imbalance model induced by cytokine inhibition. In addition to renal manifestations, some rare but biologically reasonable signals are also worthy of careful interpretation. Horner syndrome is rare, but considering the role of inflammatory mediators in autonomic nerve regulation, it may suggest that TNF-α regulation has a subtle influence on sympathetic ganglion function (34). Similarly, reports on morbid thinking suggest that changes in TNF-α-dependent neuroinflammatory pathways may affect neuropsychiatric homeostasis (35). SAPHO syndrome is characterized by inflammation of bone joints and abnormal bone remodeling, which may represent another manifestation of immune imbalance caused by cytokine blocking in bone and matrix tissue (36, 37). Although the number of cases is still limited, the mechanism consistency of these signals shows that it should not be regarded as pure accidental discovery. At the clinical level, it is recommended to be highly vigilant against these rare AEs. When the patient has atypical nervous system, psychosis, kidney (for example, elevated serum creatinine, abnormal urine routine, proteinuria), tumor or bone performance, it should be evaluated immediately, and the use of infliximab should be temporarily suspended or stopped according to careful risk-benefit assessment.

Subgroup analysis by gender showed that there were significant gender differences in AEs related to infliximab among IBD patients, which highlighted the key role of gender in drug safety evaluation. In the female subgroup, AEs were mainly concentrated in the fields of immune mediation, infection and tumor. Immune-related events, such as lupus-like syndrome, are particularly prominent, accompanied by infections including streptococcal tonsillitis. It is worth noting that rare AEs, such as SAPHO syndrome and vaginal squamous cell carcinoma, have occurred in the female subgroup, and these events have not been fully recorded in the existing drug instructions or previous literature. In contrast, AEs in the male subgroup are mainly related to cardiovascular system, nervous system and tumor. Common events include bradycardia, arrhythmia and infection, such as tuberculosis. Rare AEs include male breast cancer, radial nerve paralysis and proliferative glomerulonephritis. Further gender sensitivity analysis shows that women are more prone to immune-related signals (for example, lupus-like syndrome), while men are more prone to infection and hemodynamic abnormalities (for example, tuberculosis and bradycardia). Compared with previous reports, women’s immune-related events are consistent with existing research results (30); However, SAPHO syndrome and vaginal squamous cell carcinoma have not been recorded in the drug instructions or literature, which highlights the novelty of this study. From the perspective of mechanism, gender differences may be related to hormone regulation and immune dimorphism. Estrogen enhances B cell activation and promotes autoimmune response (38), while testosterone has a relative immunosuppressive effect, which may partly explain the phenomenon that women have higher frequency of immune-related signals and men are more prone to infection. In addition, the gender difference of TNF-α expression and its downstream inflammatory cascade may further affect the pedigree of treatment response and AEs (39). At the same time, differences in burden of complications and treatment patterns may also lead to observed differences; For example, a higher prevalence of cardiovascular risk factors in men may amplify cardiovascular signals, while a higher baseline prevalence of autoimmune diseases in women may increase the risk of immune-related events. In addition, gender differences in medical treatment behavior and adverse event reporting may introduce reporting or testing bias. Therefore, the interpretation of signal detection results from spontaneous reporting system should consider both biological mechanism and potential confounding factors.

Although cardiovascular signals and male breast cancer are rare, they may be related to the unique physiological response of men to TNF-α inhibition. Rare signals such as vaginal squamous cell carcinoma may reflect the local immune monitoring changes related to TNF-α inhibition, but this is still speculation (31). Radial nerve paralysis may represent a signal worthy of further mechanism exploration, rather than a confirmed adverse reaction (34). It is worth noting that this study is based on the database of spontaneous reports, which cannot fully adjust for potential confounding factors, such as age distribution, concomitant medication (such as corticosteroids or immunosuppressants), disease phenotype or disease severity. Therefore, the observed gender differences may reflect the combined effects of biological mechanism and residual confounding factors or reporting bias. In the future, real-world research with multivariate adjustment is needed to further verify these findings. Clinicians should adopt gender-specific monitoring strategies, focus on the immune and tumor AEs of female patients, and strengthen the assessment of cardiovascular, infection and nervous system of male patients. It should be noted that these suggestions are based on signal detection and need to be further verified in clinical research. Special attention should be paid to the atypical manifestations of rare signals to ensure early identification.

The comparison between CT group and MT group further illustrates the complexity of TNF-α inhibitors in the real world. In CT group, commonly used combination drugs, such as prednisone and azathioprine, are associated with more obvious cardiovascular and autonomic nervous system-related AEs. These findings may reflect the synergistic effect of pharmacodynamics, that is, combined immunosuppression will enhance systemic inflammatory regulation and vascular dystonia (39). Therefore, the observed signal may represent the drug interaction effect, rather than the single effect of infliximab (40). On the contrary, in MT group, immune mediation and some tumor signals seem to be more significant. Although this may indicate that infliximab monotherapy is enough to disrupt immune homeostasis and thus induce autoimmune or tumor-related events, it still needs to be cautious (30). However, previous studies generally show that the risk of malignant tumor is more common in infliximab combined with azathioprine, especially involving non-melanoma skin cancer, leukemia and lymphoma, while the evidence of monotherapy is still limited (41–43). Therefore, the tumor signals observed in MT group in this study are different from the existing conclusions, which should be interpreted with caution, because these signals may be influenced by confounding factors and inherent reporting bias in real-world data. Overall, the results of CT group may reflect that drug interaction enhances the systemic effect of TNF-α inhibitors, while MT group more directly reflects the immunomodulatory effect of infliximab (31). The cumulative effect of combined immunosuppression should be considered when interpreting the risk of malignant tumor, rather than attributing the risk entirely to a single drug. Clinically, the monitoring strategy should be adjusted according to the treatment methods: for patients receiving CT treatment, special attention should be paid to cardiovascular and malignant tumor risks; For patients receiving MT treatment, immune-related AEs and tumor risk (such as breast cancer) should be evaluated in combination with individual baseline risk factors to achieve individualized management.

AEs related to infliximab often lead to serious clinical consequences, and a considerable part of them require hospitalization or lead to death, which highlights the importance of its safety. The TTO analysis showed that the median occurrence time of AEs was 620 days; However, the shape parameter of Weibull distribution (β = 0.73) is less than 1, which indicates that there is an “early failure” mode, that is, most AEs occur in the early stage of treatment. Importantly, the relatively long median treatment time balance and the existence of β < 1 suggest that the time distribution of risk is heterogeneous, which is characterized by a high risk shortly after the start of treatment, and then gradually decreases with time, rather than most events occurring in the first month. It is worth noting that some serious AEs appeared several years after drug use, which emphasized the need to be vigilant against long-term risks. These results show that it is very important to strengthen monitoring in the first month after starting infliximab treatment, especially for potential target organs such as kidney, cardiovascular system and infection site. For patients who have been treated with infliximab for a long time, regular follow-up is very important to find the delayed serious adverse reactions. The joint analysis of TTO and WSP provides a time-dependent risk assessment framework for pharmacovigilance, which is helpful to optimize individualized monitoring strategies and improve patient safety.

Although the scope of this study is extensive, it is still limited by the inherent limitations of spontaneous reporting system. Underreporting, selective reports and incomplete clinical information may distort signal strength, especially for rare or non-serious events. Limiting the reporting target to medical professionals can improve the reliability, but may reduce the sensitivity to emerging signals. In addition, causal inference cannot be established, and unmeasured confounding factors (including disease severity, concomitant drugs and complications) may affect the results. Nevertheless, FAERS database is still of great value for hypothesis generation and early detection of unexpected safety signals (19). Future research should be included in a well-designed real-world cohort, and multivariate adjustment and mechanism research should be conducted to verify new findings, especially those related to gender differences and rare events.

## 5 Conclusion

In conclusion, based on FAERS database, this study analyzed the AE reports related to infliximab use in 80,138 IBD patients. The results showed that the burden of AE was heavy, and the incidence of AE was the highest in women and patients aged 18-64. Common AEs are consistent with known risks, such as lupus-like syndrome, tuberculosis and serum sickness. However, the study also found some rare but closely related AEs, including Horner syndrome, mesothelioma, SAPHO syndrome and vaginal squamous cell carcinoma, suggesting that there may be potential new safety problems that are not covered by the current drug labels. Gender subgroup analysis shows that women are mainly affected by immune and tumor-related events, while men experience cardiovascular and nervous system events more, which highlights the significant gender differences in drug safety. These findings suggest that gender-specific monitoring strategies should be implemented in clinical practice. In addition, corticosteroid combined with azathioprine is associated with an increased risk of autonomic nervous system and cardiovascular AEs, while monotherapy is more likely to cause immune and tumor-related events. These results emphasize the importance of FAERS database in post-marketing drug safety monitoring, and call on clinicians to be alert to emerging or gender-specific AEs. Future research should combine prospective design with molecular mechanism research to further verify these findings and clarify their potential mechanisms.

## Data Availability

The original contributions presented in the study are included in the article/Supplementary Material. Further inquiries can be directed to the corresponding authors.
